# Patients', Relatives' and Healthcare Professionals' Experiences and Perceptions of Raising Concerns About Acute Deterioration: A Qualitative Case Study

**DOI:** 10.1111/jan.70669

**Published:** 2026-07-08

**Authors:** Aidín McKinney, Donna Fitzsimons, Bronagh Blackwood, Jennifer McGaughey

**Affiliations:** ^1^ School of Nursing & Midwifery Queen's University Belfast, Medical Biology Centre Belfast UK; ^2^ School of Medicine, Dentistry and Biomedical Sciences Wellcome‐Wolfson Institute for Experimental Medicine, Queen's University Belfast Belfast UK

**Keywords:** acute deterioration, Martha's rule, patient and family‐initiated escalation of care

## Abstract

**Aim:**

To explore adult patients', relatives', and healthcare professionals' experiences of acute deterioration and perceptions of patient/family involvement in raising concerns.

**Design:**

A collective case study underpinned by implementation science.

**Methods:**

Individual and focus group semi‐structured interviews were conducted with patients, relatives and healthcare professionals who had experience of deterioration across two urban hospitals. The interview schedule was informed by the Theoretical Domains Framework of behaviour change and a concurrent review of patient notes was undertaken to contextualise the deterioration experience. Data was analysed using thematic analysis.

**Findings:**

Between March and October 2019, 12 patients, 11 relatives, and 32 healthcare professionals were interviewed. Findings revealed that patients and relatives can detect deterioration, and they highlighted challenges and benefits in their involvement in raising concerns. Perspectives from all stakeholders highlighted an urgent need to provide better support and mechanisms for patients and relatives in escalating deterioration. These include the need to co‐design innovative interventions that focus on informing patients/relatives of the main signs of deterioration, key steps to take when calling for help, and their role in the process.

**Conclusion:**

Understanding the perceived challenges and benefits of patient and family‐initiated care escalation provides a strong theoretical and practical foundation for designing and implementing an effective intervention.

**Impact:**

The findings highlight key processes and intervention components essential for developing a patient and family‐initiated escalation of care intervention.

**Implications for the Profession and/or Patient Care:**

This insight will support the co‐design and implementation planning of an intervention aimed at preventing or reducing deterioration by empowering patients and their families to escalate concerns.

**Reporting Method:**

The COREQ checklist was used as a guide for reporting the study.

**Patient or Public Contribution:**

Patients, relatives and healthcare professionals were involved in the co‐design of the study.

## What Does This Paper Contribute to the Wider Global Clinical Community?

- While patient and family involvement in raising concerns about acute deterioration is increasingly advocated, robust research that utilises theoretical underpinnings to explore all stakeholders' views and understand context remains scarce.
- Patients, relatives and healthcare professionals in this study highlight an urgent need for clear mechanisms to help families recognise and/or report signs of deterioration, understand steps for escalation and know their role in the process.
- These findings provide a strong theoretical and practical basis for developing and implementing effective escalation interventions.

## 1 Introduction

Failures in the recognition and response to clinical deterioration continue to be a significant area of concern (Welch et al. 2025). The contribution that patients and/or families can make to detect and manage acute deterioration is increasingly being highlighted and several patient and family‐initiated escalation of care interventions have been implemented internationally in Australia, United States of America (USA), New Zealand (NZ) and the United Kingdom (UK) (Dwyer et al. 2020; Odell 2019). More recently, this has received increased attention in the United Kingdom with “Martha's Rule” being introduced in a number of hospitals in England (NHS England 2025). However, research‐based evidence that has sought the perspective of patients, relatives and healthcare professionals (HCP) to inform the co‐design of these interventions has to date been limited.

## 2 Background

It is estimated 10% of patients experience clinical deterioration during their hospital stay (Donaldson et al. 2014). To address the problem, rapid response systems and protocolised healthcare interventions have been introduced in hospitals internationally even though research evidence suggests that these processes do not always work due to cultural, behavioural or organisational factors (McGaughey, O’Halloran, Porter, and Blackwood 2017; McGaughey, O’Halloran, Porter, Trinder, and Blackwood 2017; Smith et al. 2021). The contribution that a patient and/or family‐initiated escalation of care intervention can make in recognising and responding to acute deterioration is increasingly being explored as a supplementary alternative (Ngoya Ntumba et al. 2023). These interventions enable worried patients and/or relatives to independently escalate concerns to healthcare professionals to assess and respond to clinical deterioration (Albutt et al. 2017). Their aim therefore is to provide an additional safety mechanism for acutely unwell patients to be identified, referred and managed earlier, rather than deteriorating further and developing potentially more complex care needs (Bucknall et al. 2021).

However, the implementation of existing family‐initiated escalation of care interventions in adult hospitals has largely been paternalistic, having been developed by healthcare professionals (King et al. 2019) or with limited input from external consumer organisations (Hueckel et al. 2012; Odell et al. 2010). There has also been little research‐based stakeholder input that has considered the extent to which patients and relatives can recognise acute deterioration or identified intervention preferences, particularly from the adult setting (Gill et al. 2016; Teo et al. 2025; Vorwerk and King 2016). In the paediatric setting there is some evidence of clinician as well as family opinion on the barriers and facilitators to developing or implementing escalation systems (Gill et al. 2018; Gill et al. 2019; Gill et al. 2025; Marufu et al. 2025; Mills et al. 2025), but only more recently have studies in the adult setting considered the patient/relative (Bucknall et al. 2024; King et al. 2019; Strickland et al. 2019; Yu et al. 2022) or the healthcare professional (Albutt et al. 2020; King et al. 2024; Thiele et al. 2022) perspective. However, given that these studies have largely been conducted in countries that have established patient/family‐initiated escalation interventions in place, it would be of value to more fully explore research in this area from a more rounded perspective by considering the views of all key stakeholders within a similar setting. This is also of importance given that there remains a significant gap in relation to understanding the contextual factors that may influence the introduction of such systems (Subbe et al. 2024; Sutton et al. 2025). As is recognised in the implementation science literature, although a particular intervention may be acceptable, affordable, and operationally feasible for one healthcare setting, it may not necessarily fit another (Goodyear‐Smith et al. 2015). The need to therefore understand various stakeholder perspectives and context and to utilise this information to co‐design patient/family escalation of care interventions that are “fit for purpose” has increasingly been recommended (Bucknall et al. 2024; King et al. 2024; Teo et al. 2025).

As a result, this is the first known research study in the adult setting that aimed to utilise a case study design to collectively consider the perspective and experiences of patients, families, and healthcare professionals (prior to developing or implementing patient/family escalation of care) and use this to co‐design a prototype intervention and implementation plan as part of a larger study (McKinney et al. 2020).

## 3 The Study

### 3.1 Aim/s

This study aimed to explore patients', relatives' and healthcare professionals' experiences of acute deterioration and perceptions of patient/family involvement in raising concerns in the acute adult hospital setting.

## 4 Methods

### 4.1 Design

This study comprises phase 2 of a large co‐design project: the study protocol was previously published (McKinney et al. 2020). The study approach was underpinned by implementation science and the Medical Research Council's (MRC) guidance for the development of complex interventions (Craig et al. 2019). In this phase 2 study, a collective case study design involving semi‐structured individual and focus group interviews with various stakeholders from two hospital settings was employed. Case study methodology was chosen because complex interventions are closely intertwined with the contexts in which they are implemented. This approach enables an in‐depth, multifaceted examination of a phenomenon within its real‐world setting and is considered particularly valuable for understanding complex issues in healthcare research (Boblin et al. 2013; Crowe et al. 2011).

The collective case study design adopted involved the investigation of multiple cases to develop a broader and more comprehensive understanding of a particular phenomenon (Baxter and Jack 2008; Stake 1995). Examining multiple cases allows the phenomenon to be explored from different perspectives rather than through a single lens (Yin 2018). Consequently, collective case studies facilitate the exploration of individuals, organisations, and the multivariate factors and processes that influence the phenomenon of interest. As such, they represent a valuable methodological approach within health sciences research for informing both theory development and intervention design (Baxter and Jack 2008).

### 4.2 Sample/Participants

The case study approach involves collecting evidence from multiple sources (Stake 1995; Yin 2018). Using purposive sampling across two hospital sites, data were collected from patients and relatives with varying experiences of deterioration, as well as from different healthcare professionals. In addition, patient case notes were reviewed.

The findings were structured using the consolidated criteria for reporting qualitative research (COREQ) to enhance transferability of the findings (Tong et al. 2012).

### 4.3 Setting and Recruitment

#### 4.3.1 Sample/Participants

The study was conducted in one acute care hospital in Northern Ireland and one in the Republic of Ireland. A purposive sampling method was employed to recruit participants who had experienced patient deterioration and escalation of care. Patients and families were identified for participation by the Critical Care Outreach Teams (CCOT) if they met the following criteria: the patient had triggered an urgent medical and/or CCOT response; the patient had a CCOT review; were 18 years of age or over; spoke English; and were physically and cognitively able to communicate. Patients and families were excluded if the patient was approaching end‐of‐life, had a cognitive/physical impairment that affected their ability to communicate, and families of deceased patients. Healthcare professionals (HCPs) from the acute wards and critical care settings with greater than 6 months of acute care experience were eligible for inclusion. The eligibility criteria for the purposive sampling strategy were designed to ensure variation in stakeholder experiences was captured. Recruitment continued until data saturation was reached, defined as the point whereby no new insights emerged from the last few interviews (Polit and Beck 2017).

### 4.4 Data Collection

Face‐to‐face and telephone semi‐structured individual and focus group interviews were conducted by the first author who was female and a registered nurse lecturer with previous experience of carrying out qualitative research. The first author was not affiliated in any way with either of the hospital sites, of which participants were informed. The semi‐structured interview schedule was informed by the Theoretical Domains Framework (Atkins et al. 2017; Cane et al. 2012), previous interview schedules by Rainey et al. (2015) and Gill et al. (2018) and a systematic review of patient and family concerns about clinical deterioration (McKinney et al. 2021) and had been pilot tested prior to data collection (File S1). The face‐to‐face interviews were conducted in a private room in the hospital or in the participants' own home, according to participant preference. A notetaker was present in the focus group interviews. Interviews were audio recorded and were 45–70 min in length. A review of the patients' medical and nursing records/notes was conducted to inform the interviews and contextualise the period of clinical deterioration and referral or escalation process. Field notes and a reflective journal were kept to document initial interpretations of the data and plan data collection.

### 4.5 Data Analysis

Interviews were transcribed verbatim by an independent transcriber and entered into NVivo 11 (QSR International 2012). Data were analysed inductively using Braun and Clarke's six step thematic analysis approach (Braun and Clarke 2012). Although the Framework Method (Pope et al. 2000) was considered as an analysis approach due to the TDF partly informing the interview guide, a key aim of the study was to explore patients' and relatives' experiences of acute deterioration. It was therefore considered important that analysis remained grounded in the data, and that themes could later be mapped onto the TDF domains if deemed appropriate. This approach is consistent with the perspective of Crowe et al. (2011), who caution against forcing findings into predetermined frameworks. By avoiding early reliance on such a framework, the researcher aimed to remain more open to identifying new and emerging themes. Nevertheless, it is important to acknowledge that the use of a theoretical framework to inform the study may have limited the extent to which analysis could be entirely neutral.

The steps determining the themes are shown in File S2. The review of case notes was analysed by classifying the reason for the deterioration, the Early Warning Score (EWS), whether patient or family raised concerns were documented and how it was managed to provide context to the deterioration experiences.

### 4.6 Ethical Considerations

Study approval was granted by the research ethics committees of Northern Ireland (Ref: 19/EE/0061) and the Republic of Ireland (Ref: C.A.2126) in March 2019. Participants provided informed consent and a distress protocol was in place.

### 4.7 Rigour and Reflexivity

A number of methodological strategies were incorporated to ensure credibility, dependability, confirmability and transferability to increase the ‘trustworthiness’ of the findings (Nowell et al. 2017; Lincoln and Guba, 2000).

Creditability was achieved by interviewing participants from a broad range of ages and backgrounds on the phenomenon of interest, thereby gathering multiple perspectives to ensure that a complete picture was portrayed (Houghton et al. 2013). The use of digital recording, verbatim typing of transcripts, and participant verification of interview summaries also enhanced credibility.

To enhance confirmability and reduce bias, a second researcher (JMcG) independently reviewed an initial 25% of the transcripts alongside the primary researcher, identifying emerging NVivo codes and themes. In addition, all members of the research team had access to the full set of transcripts, enabling ongoing discussion, refinement, and agreement of themes during regular research meetings, thereby supporting the dependability of the analysis process.

Confirmability was also addressed through reflexivity by using a reflective journal to document thoughts, biases, reflections and assumptions, ensuring that the research team remained cognisant of their professional backgrounds and the potential that this could have to influence data interpretation.

Transferability was supported through detailed reporting of the research process, rich contextual descriptions of participants, settings, and findings, and the inclusion of participant quotations. This allows readers to assess the applicability of the findings to other settings or populations.

## 5 Findings

### 5.1 Participant Characteristics

Between March and October 2019, 12 patients (Site A: 6; Site B: 6), 11 relatives (Site A: 6; Site B: 5) and 32 HCPs (Site A: 17; Site B: 15) participated in individual or focus group interviews. HCP included managers (6), doctors (4), ward nurses (14), and CCOT nurses (8) with acute care experience ranging from 1–32 years to provide a range of acute care professional perspectives. Patient EWS trigger scores ranged from 7–11, indicating significant acute deterioration. In total, 6 patients required transfer to High Dependency or Intensive Care. Reasons for transfer consisted of the need for ventilatory support, close monitoring and/or high levels of oxygen therapy. Reasons for clinical deterioration varied, although infection/sepsis was identified as being a main reason for deterioration in both case sites. Only one documented note of concern by a relative was reported in the case notes of one patient. The numbers and breakdown of patient participants and deterioration occurrences from both case sites are shown in Table 1.

**TABLE 1:** Patient demographics.

Participant ID No	Gender	Age	Diagnosis	Reason for CCOT referral	EWS	Documented concerns by patients or relatives in notes	Transferred to ICU/HDU
Case site A							
33	Male	62	Pneumonia	Increased SOB	8	No	No
34	Male	44	Pancreatitis	Sepsis	8	No	No
35	Female	31	Diabetic ketoacidosis, Leg infection	Sepsis	8	Yes	Yes
36	Female	62	Sepsis secondary to cellulitis	Sepsis	10	No	No
37	Female	75	Cholecystitis	Low BP	9	No	Yes
38	Female	33	Sepsis‐bacteraemia	Sepsis	7	No	No
Case site B							
45	Male	67	Pneumonia Sepsis	Sepsis	9	No	Yes
46	Male	79	Pancreatitis Fluid overload	Increased SOB	8	No	Yes
47	Female	62	Chest infection Sepsis	Sepsis	9	No	Yes
48	Female	44	Breast cancer, Sepsis	Respiratory decompensation	9	No	No
49	Female	60	Multiple myeloma	Increased SOB	11	No	Yes
50	Female	63	Pneumonia, sepsis	Sepsis	8	No	No

Abbreviations: BP, blood pressure; SOB, shortness of breath.

### 5.2 Qualitative Interviews – Themes and Sub‐Themes

Three main themes were identified: (1) experiences of deterioration, (2) challenges in calling for help, and (3) ways to support. The three themes and seven associated subthemes are presented in Table 2. The thematic map is depicted in File S2 and additional supporting quotes from the analysis are also included here.

**TABLE 2:** Case study themes and subthemes.

Themes	Sub‐themes
1. Experiences of deterioration	Knowing the patient Being heard
2. Challenges in calling for help	Knowledge/skill deficits Personal and role conflict factors Resource/organisational deficits
3. Ways to support	Signposting Enhancing engagement

#### 5.2.1 Experiences of Deterioration

##### 5.2.1.1 Knowing the Patient

Knowing the patient (or self) best was highlighted by most patients and relatives across both case sites as a significant factor in their ability to identify a change in their condition. Relatives particularly described how it is only because they, as relatives, know the person so well and over a wider time frame that they can pick up on subtle cues that may never be fully appreciated by healthcare staff. Most healthcare professionals similarly agreed that patients and/or relatives know the patient best and provided examples whereby patients and/or relatives were able to alert them to serious changes in their condition, sometimes before they had been identified by staff on EWS systems.

Relatives often know a patient much better than we do. We see them for perhaps a snapshot of fifteen minutes… Whereas a relative will know what they're like when well and probably more readily sense that something is changing, potentially before NEWS and things we look at to see if someone is changing (Ward Doctor, A [04]).

In contrast a small number of healthcare professionals (night manager, a critical care doctor and a few ward nurses) were less certain about relatives' ability to contribute to detecting deterioration, questioning whether the relatives of adult patients have the same degree of intuition that a child's parents may have. However, this viewpoint was not commonly shared.

##### 5.2.1.2 Being Heard

Across both case sites, several patients and their relatives shared experiences of reporting deteriorations and feeling heard. However, others felt that their concerns were dismissed or that they were talked down to. As a result, they often felt their opinions were disregarded, especially when no vital sign abnormalities were apparent. These individuals spoke about the emotional impact of this, expressing feelings of extreme distress, frustration, and powerlessness. Moreover, some relatives mentioned that to be heard it required significant resilience and persistence on their part. This perspective was also echoed by certain healthcare professionals—including ward nurses, a critical care manager, and a ward doctor—who reflected on their own experiences as relatives and noted the difficulty of being listened to, even with insider knowledge.

I was met with a wall. And really it took… barrier breaking to say, can somebody listen? (Ward nurse 2, FG2, B [30])

Additionally, critical care doctors, managers, and outreach nurses believed that some healthcare professionals may not always fully listen to or take seriously the concerns of patients and relatives regarding deterioration. They all felt that this valuable insight is often underutilised.

#### 5.2.2 Challenges in Calling for Help

##### 5.2.2.1 Knowledge and Skills Deficits

Patients and relatives at both case sites expressed difficulty in knowing who to approach or how to seek help when concerned about deterioration, aside from the nurse on the ward. A small number reported that they reverted to raising a complaint, feeling it was the only option left to enable their concerns to be heard. Healthcare professionals, including ward managers, nurses, doctors, and critical care outreach nurses, also acknowledged that it can be challenging for patients and relatives to understand how to request assistance, especially when they feel unheard by their care team. Interestingly, these challenges were even reported by relatives who were healthcare professionals.

I wasn't aware that I could ask somebody else. I just felt it was only the nurse who was on (Relative 2, B [52]).

##### 5.2.2.2 Personal and Role Conflict Factors

Many patients and relatives additionally expressed a significant reluctance to raise concerns about deterioration with staff, with a large number expressing fears that by doing so, they might overstep the healthcare professionals' role. Additionally, some relatives described the hospital experience as intimidating and felt that it challenges the ability of any patient or relative, regardless of their confidence, to voice concerns. Numerous healthcare professionals, including ward nurses, critical care nurses, and critical care consultants, agreed that hierarchical imbalances persist which contribute to an intimidating environment and difficulties in speaking up, especially among the older generation.

Some people won't be confident enough to raise it… and it is probably slightly older patients who are maybe very much, the doctor or nurse knows best. And they'll not want to make a fuss (Critical Care Doctor, A [02]).

##### 5.2.2.3 Resource/Organisational Deficits

Resource and organisational shortcomings were also identified as factors that made speaking up more difficult. Most patients and relatives described a rushed, busy atmosphere on the ward, leading them to hesitate about adding to the workload. Healthcare professionals also acknowledged the staffing pressures they face, noting how challenging it can be to be as available to patients and relatives as they would like. Additionally, they agreed that patients and relatives were also extremely aware of the staffing pressures they face and acknowledged that this “put them off” seeking help early. Furthermore, a critical care doctor, ward nurses, critical care outreach nurses, and a ward doctor felt that the organisation did not provide sufficient support for raising and addressing real‐time deterioration concerns. One critical care consultant highlighted that while there is an established complaints process, there are currently no policies/processes that deal with real‐time deterioration concerns as they arise.

…There's certainly a well‐defined complaints structure… But it is not the right structure to deal with that sort of concern that things aren't going the way they should be… (Critical Care Doctor, A [02]).

#### 5.2.3 Ways to Support

##### 5.2.3.1 Signposting

Most patients and relatives felt it would be helpful to have clearer guidance on how to seek help when concerned about deterioration, including information on who to approach, the types of concerns that warrant escalation, and clarification on whose role it is to detect and escalate care. They also suggested that providing details on key signs and symptoms of deterioration could be valuable, as they highlighted that there can at times be a tendency to question whether symptoms are serious enough to require assistance. Most patients and relatives identified that they would initially attempt to speak to their own nurse or doctor about their concerns, however they also proposed the idea of having access to a healthcare professional outside their immediate ward team, someone who could listen to and support them with concerns about deterioration, as being of merit. A few believed this should be an experienced professional who could coordinate with other staff on their behalf. Some were aware of the critical care outreach team and thought they could fulfil this role, while others reported feeling intimidated by the idea of contacting such a specialist team. These individuals expressed that they would feel more comfortable reaching out to someone in a neutral role who could communicate with the rest of the healthcare team, including the outreach team, on their behalf.

I think that could be a bit scary, for someone… and you don't want them ringing the critical care nurses because the person looks a bit more sleepy that day… maybe someone who had a more neutral role (Patient 6, A [38]).

Healthcare professionals (ward managers, ward nurses, critical care outreach nurses and a critical care doctor) agreed that there is a need to provide clearer guidance to patients and relatives on how to seek help, and who to go to. They also felt that the inclusion of key signs of deterioration would help patients and relatives verify deterioration and discuss it with staff.

Additionally, some ward nurses and a critical care manager felt that an emphasis should be placed on encouraging relatives or patients to raise any concerns ‘if something did not feel right’.

Again, I am going back to intuition. This isn't about them having assessment skills. It's about them intuitively knowing their family member (Critical Care Manager, B [18]).

Healthcare professionals varied in their opinions regarding facilitating patients/relatives to have access to a healthcare professional outside their own care team. Some, including a hospital at night manager and several ward nurses, felt that while patients and relatives might recognise signs of deterioration, they may lack the expertise to assess the severity and escalate the issue appropriately. On the other hand, a ward doctor, a critical care doctor, a ward manager, some ward nurses, and most critical care outreach nurses felt that there may be value in allowing patients and relatives to escalate concerns beyond the ward team, especially when staffing levels are limited. Reflecting the patients and relatives' perspective, most healthcare professionals believed that this role should be filled by an experienced healthcare professional with strong communication and advanced acute care assessment skills. They noted that while critical care outreach nurses possess these skills, they felt that other staff, such as Emergency Department (ED) nurses and night managers, also have similar assessment capabilities and could therefore expand the resource pool to draw from.

I come from a critical care background, so I keep pulling from there. But it doesn't necessarily have to be an ICU nurse… you could argue ED nurses, are also very good at identifying acutely unwell people (Critical Care Manager, B [18]).

##### 5.2.3.2 Enhancing Engagement

Most patients and relatives also felt that healthcare professionals should proactively ask patients and families whether they have any concerns about deterioration. A few healthcare professionals, including a critical care consultant, ward nurses, and critical care nurses, agreed that it would be beneficial to create more deliberate opportunities for patients and relatives to raise concerns or problems they were experiencing. Some suggested that the concerns of relatives should also be included as a scoring criterion in the Early Warning Score (EWS).

I think just… asking them how they are… And be a bit more proactive that way. Come and find you instead of the other way round (Relative 4, A [42]).

## 6 Discussion

This study provides new insights into patients' and relatives' ability to detect acute deterioration from the combined perspectives of patients, relatives and HCPs. There was unanimous agreement that patients and relatives know the patient best and have valuable and legitimate insights into the patients' condition which aligns with findings from other studies (Bucknall et al. 2024; Guinane et al. 2018; McKinney et al. 2021; Odell et al. 2010; Strickland et al. 2019). However, this study also additionally details the perspective of healthcare professionals, which has received less focus in the adult setting. A particularly notable finding was that both relatives and healthcare professionals shared examples where relatives reported on acute deterioration episodes that were not initially apparent to healthcare professionals but were nevertheless valid concerns. This finding aligns with Albutt et al. (2020) who also found that healthcare professionals recognise relatives' concern as a frequent and valid predictive indicator of deterioration. This further emphasises the importance of viewing patients and relatives as knowledgeable partners in care (Bucknall et al. 2021).

The challenges some patients/relatives face when detecting deterioration and trying to call for help were also highlighted in this study, further reinforcing findings from other research (Dwyer et al. 2020; Gill et al. 2018; King et al. 2019; Rainey et al. 2015). As in previous studies, this difficulty was attributed to a lack of knowledge about how to navigate the system or a reluctance to speak out due to concerns about adding to staff workload (Guinane et al. 2018; Rainey et al. 2015; Strickland et al. 2019; Thiele et al. 2025). Additionally, perceptions of the roles of patients, relatives and healthcare professionals influenced their ability to speak up, a challenge also noted in other studies (Dwyer et al. 2020; Guinane et al. 2018; King et al. 2019; Rainey et al. 2015; Strickland et al. 2019). This paints a picture of a busy, stretched work environment that may result in staff being less able to always monitor for or detect deterioration (Subbe et al. 2024). Perceptions of role and hierarchy in healthcare settings have also been shown to influence communication and can impact not only on what is being said but how comfortable people feel about speaking up (Thiele et al. 2022). To address these challenges, patient and family escalation of care interventions should address these power imbalances and promote and encourage clear lines of communication.

An additional interesting finding to emerge from this study was that patients and relatives noted that they can lack confidence that they have the necessary knowledge or skills to fully appreciate the symptoms of deterioration to validate their concerns. This mirrors findings from Albutt et al. (2020) study, where healthcare professionals reported that relatives might misjudge the seriousness of symptoms due to a lack of clinical knowledge. However, while Albutt et al. (2020) study reported that staff felt it would not be appropriate to educate patients and relatives on symptoms of deterioration, this study differed, as many patients, relatives and healthcare professionals felt that this would be very worthwhile. This suggests that patients and relatives are more willing to acquire knowledge about their health, particularly in relation to acute changes than previously recognised, and this should be acknowledged and addressed at organisational level (WHO 2021).

While educating members of the public on symptoms of acute deterioration is not uncommon in the community (Thiele et al. 2020), research on educating hospital patients and relatives in this area is limited. The only previous study identified was by See et al. (2014) which explored healthcare professionals providing education to hospitalised patients on recognising worsening symptoms and reported that they felt this approach had merit. Our findings add to this evidence and uniquely further explore the value of this education as part of a patient and family‐initiated escalation of care intervention.

Stakeholder preferences regarding preferred escalation pathways have also received limited attention in previous research (King et al. 2019). Our findings highlighted that patients, relatives, and healthcare professionals reported a preference to escalate concerns initially to their own care team, believing that clearer signposting would improve this process and reduce the need for further escalation. However, all stakeholders acknowledged that there may be occasions when patients and relatives may need to escalate concerns outside their own team. This is echoed and reinforced by the numerous, global examples where families tried to raise deterioration concerns and were not listened to (Bucknall et al. 2021; King 2010; Mills 2022). Unlike many other patient/family‐initiated escalation of care interventions that encourage direct escalation to the CCOT (Brady et al. 2015; Odell et al. 2010), patients/relatives and clinical professionals from this study were not as universally supportive of solely utilising this route, even when CCOTs were available. Instead, having the option to escalate concerns to a qualified healthcare professional with acute assessment skills, whose role was to liaise with families and/or with the CCOT if required, was considered to be less daunting and more acceptable by some. This finding aligns with King et al. (2019) who found that having a patient liaison or advocate with advanced professional knowledge was an acceptable alternative for escalating care when concerns remain unresolved. Some healthcare professionals, in this study, drawing on their experiences as relatives of deteriorating patients, also shared this reluctance to escalate directly to the CCOT. This echoes the findings from the National Patient Safety Foundation (2014) which identified that even those with insider knowledge of the healthcare system can feel intimidated to ask questions or seek help. Furthermore, the limited availability of 24/7 CCOTs in both case sites further emphasises the importance of considering local contexts when adopting interventions from other settings, as what works in one environment may not be feasible in another (Welch et al. 2025).

Finally, this study identified that while all participant groups agreed that patients and relatives can detect deterioration and should have a role in escalating concerns, there were variations regarding the extent to which they believed patients and relatives could or should be involved in this process. As seen in other studies, this suggests that current organisational culture does little to encourage patient and relative involvement in this area (Sutton et al. 2025). The findings suggest that future interventions should carefully consider these insights to ensure effective and meaningful involvement of patients and relatives in escalation processes.

## 7 Strengths and Limitations

This research study contributes to the growing international body of work that is focusing on patient and family involvement in the detection and escalation of acute deterioration. In particular, by utilising a theoretical informed, case study approach, it offers a unique perspective into the ability of patients and relatives to detect deterioration and preferences for involvement in escalation of care from the collective perspective of patients, relatives and HCP's. This approach also helps to extend insights into the contextual factors that need to be considered prior to developing an intervention. The case study was conducted at two case sites in Northern Ireland and the Republic of Ireland, each with distinct healthcare systems. Additional input from participants in other contexts could have enriched the perspectives gathered. However, expanding to more sites was not feasible due to time, resource constraints, and the limited number of case sites with a Critical Care Outreach Team (CCOT) particularly in the Republic of Ireland. Nonetheless, the findings from both case sites were consistent, regardless of the healthcare jurisdiction.

It should also be noted that all patient participants in this study exhibited significant clinical deterioration, as indicated by their high EWS scores. This was not due to intentional sampling; as all patients referred to the outreach team, including those less severely ill, were invited to participate by the gatekeeper. However, during the data collection period, only patients with severe deterioration were recruited. This incidental sampling should be considered when interpreting the study findings, as the predominance of severely unwell participants may have influenced the results.

## 8 Implications for Policy, Practice and Research

This study provides further evidence that concerns raised by patients, families, or carers are vital indicators of clinical deterioration. It also further emphasises that within the healthcare setting, two expert teams co‐exist—patients and their relatives, and healthcare professionals. However, there remains a need for this perspective to be more fully recognised and embraced by both parties, as well as at an organisational level.

The findings underscore the urgent need for models of patient and family escalation systems to be adopted across all healthcare settings to improve outcomes. In particular, they emphasise the importance of more actively enhancing engagement opportunities to promote listening and communicating with patients and families regarding clinical deterioration concerns, in line with the principles outlined in Martha's Rule (NHS England 2025). The study also offers valuable insights into how patients and families can be better supported to escalate concerns. This includes the need to develop more effective and accessible communication pathways and adopt approaches that actively and more explicitly promote their meaningful involvement. Providing education on key signs of deterioration may also be beneficial, but the modality of communication to promote understanding and access must be carefully considered. Although ward posters and other informal materials are commonly used, it has been suggested that these are often low‐value and insufficient on their own, partly due to overuse and limited consideration of adult literacy (Soong and Shojania 2020). However, given that it is estimated that approximately one in five adults in Northern Ireland and Ireland have poor literacy skills (National Literacy Trust 2021), consideration of strategies to improve widespread understanding is important. Consequently, efforts to improve public and patient understanding of clinical deterioration should prioritise clear, accessible language and co‐designed communication strategies developed collaboratively with patients, relatives, and healthcare professionals.

The findings also emphasise the importance of tailoring interventions to local context and organisational characteristics. However, there remains a notable lack of robust research applying implementation science approaches to co‐design and evaluation of systems that are both effective and contextually appropriate. Addressing this gap represents an important priority for future research and service development.

### 8.1 Future Research

This study focused on the initial development phase of the MRC framework for the development of complex interventions (Craig et al. 2019), which advocates engagement with key stakeholders and appreciation of the real‐life setting. These findings can be utilised to inform the initial part of a co‐designed intervention as part of a larger research study.

## 9 Conclusion

This study highlights that patients and relatives can detect acute deterioration and overall, patients, relatives, and healthcare staff within the island of Ireland are supportive of patient/family involvement in the recognition and escalation of acute deterioration. However, current mechanisms do not currently facilitate this process. Patients, relatives and healthcare staff collectively recognised the urgent need for an intervention that addresses this gap. Additionally, a clear picture of the main areas that require focus when developing the intervention emerged and emphasised that the intervention should target patient/relative knowledge and skills, professional role and identity, as well as beliefs about capabilities and consequences. Furthermore, the study provided insights into the challenges that may influence the development and implementation of family escalation systems, including personal and organisational bias, the capacity of the system to respond to escalation concerns and service user insecurities regarding raising concerns. While lessons from international family escalation systems may help inform future intervention development, a deeper understanding of the local context, the role of co‐design, and organisational willingness to support and sustain such systems is equally important. These findings can be used to develop a theory‐based intervention to improve patient and relative involvement in the detection and escalation of acute deterioration.

## Funding

The Health Service Executive of Ireland has funded this research study as part of the PhD Scholars Programme in the Programme for Health Service Improvement (PHSI). The HSC R&D Division Northern Ireland (NI) provided funding to support Patient and Public Involvement (PPI) in the study.

## Ethics Statement

Ethical permission was obtained for this study in March 2019 from the research ethics committees of Northern Ireland (Ref: 19/EE/0061) and the Republic of Ireland (Ref: C.A.2126).

## Conflicts of Interest

The authors declare no conflicts of interest.

## Supporting information


**File S1:** Interview Guides.

**File S2:** Data Analysis for Stakeholder Interviews.

## Data Availability

The data that support the findings of this study are available on request from the corresponding author. The data are not publicly available due to privacy or ethical restrictions.
