# Supplementary material for: Patients', Relatives' and Healthcare Professionals' Experiences and Perceptions of Raising Concerns About Acute Deterioration: A Qualitative Case Study
Source: J Adv Nurs. 2026 Jul 8;82(12):10379–89. doi: 10.1111/jan.70669 (PMC13752666; doi:10.1111/jan.70669)
Supplement: Supplementary file 1 [file JAN-82-10379-s001.docx]

**Supplementary File 1: Interview Guides**

**Semi-structured Interview Guide for Patients/Relatives**

**Welcome/Introduction**

- Welcome to this focus group/individual interview.
- Introduce self.
- The purpose of this interview is to hear about your experiences of becoming more unwell (or your relative becoming more unwell) while in hospital and your views about an intervention or resource that might allow patients/relatives to have a greater voice in calling for help if you were concerned about a sudden change in your (or your relatives’) condition.

**Consent**

- Inform participants of the need for written consent and ask if any further clarification of the interview process is required
- Obtain individual consent from each individual participant.

| Main topic areas | Questions |
| --- | --- |
| Open invitation statement and general questions | Tell me a little about your (or your relatives) experience while in hospital?  What can you tell me about your experiences of being unwell? (or your relative)  What can you tell me about what you did or said if you felt that your (or your relatives’) condition had changed? |
| 1. Knowledge | How do you feel about a resource that may help patients and relatives to have a greater voice in calling for help if they felt that their condition had become more unwell and they were concerned?  What type of format do you imagine, if any, that this might take? |
| 2. Skills | Can you describe what skills, if any, that you feel you would need to have if you wanted to call for help if you felt that you (or your relative) became more unwell and were concerned? |
| 3. Beliefs about capabilities | How easy or difficult do you believe it would be for you to raise your concerns or call for help? |
| 4. Beliefs about consequences | What do you believe might happen if you raised your concerns or called for help?  What might the benefits be of patients/relatives having a role to play in calling for help? What might the consequences be? |
| 5. Social/professional role and identity | Can you tell me a bit about how you feel about patients/relatives having a role in recognising deterioration or changes in their condition and calling for help?  How about the healthcare professionals’ role?  How do you feel staff might feel about you raising your concerns or calling for help? |
| 6. Memory, attention and decision processes | What might help you to make a decision for you to call for help?  What might help you/prevent you in remembering how to go about calling for help? |
| 7. Goals | What would motivate you to call for help?  What would hinder/prevent you from calling from help? |
| 8. Intention | What do you think might happen if a scheme like this was put into place? |
| 9. Reinforcement | What incentives might there be for you to become involved with a scheme like this?  What would put you off becoming involved in a scheme like this? |
| 10. Optimism | How valuable, or not do you feel a scheme like this might be? |
| 11. Environmental context and resources | Can you tell me about the practicalities of what might be required for this scheme to work in practice? Such as resources? |
| 12. Social influences | Can you tell me more about who you feel supports you if you have concerns? What support structures do you feel already exist if you wish to raise concerns about a change in condition? |
| 13. Emotion | How might calling for help affect you? Can you describe any fears/anxieties that you might have regarding calling for help? |
| 14. Behavioural regulation | Can you describe what might encourage you to become more involved in a scheme like this? What might put you off? |
| Prompts | Could you tell me about …  How do you feel about …  What does it mean …  What do you think …  Can you tell me a bit more about …  How do you see … |

*Adapted from Interview schedules by Rainey et al (2015) and Gill et al (2018) and is based on the Theoretical Domains Framework (14 Domains) (Atkins et al 2017; Cane et al 2012)*

**Semi-structured Interview Guide for Healthcare Profession**als

**Welcome/Introduction**

- Welcome to this focus group/individual interview.
- Introduce self.
- The purpose of this interview is to hear about your experiences while caring for a patient who deteriorated in hospital and your views about an intervention or resource that might allow patients/relatives to have a greater voice in calling for help if they were concerned about a sudden change in the patients’ condition.

**Consent**

- Inform participants of the need for written consent and ask if any further clarification of the interview process is required.
- Obtain individual consent from each individual participant.

| Main topic areas | Questions |
| --- | --- |
| Open invitation statement and general questions | Tell me a little about your experiences, if any, of patients/relatives raising concerns while in hospital if they felt their condition was deteriorating? |
| 1.Knowledge | How do you feel about a resource that may help patients and relatives to have a greater voice in calling for help in they felt that their condition had become more unwell and they were concerned?  What type of format do you imagine, if any, that this might take? |
| 2.Skills | Can you describe what skills, if any, that you feel patients/relatives would need if they were to become involved in a scheme like this?  What skills if any would you need? |
| 3.Beliefs about capabilities | How easy or difficult do you believe it would be for healthcare professionals to become involved in a scheme like this? |
| 4.Beliefs about consequences | What do you believe might happen if a scheme like this was introduced into practice?  What might the benefits be of patients/relatives having a role to play in calling for help? What might the consequences be? |
| 5.Social/professional role and identity | Can you tell me a bit about the patients/relatives role in recognising deterioration or changes in their condition and calling for help?  How about the healthcare professionals’ role? |
| 6.Memory, attention and decision processes | What would need to be remembered or paid attention to in developing a resource like this? |
| 7.Goals | What would motivate you to become involved or not in a scheme like this?  What would hinder/prevent you from becoming involved in a scheme like this? |
| 8.Intention | What do you think might happen if a scheme like this was put into place? |
| 9.Reinforcement | What incentives might there be for you to become involved with a scheme like this?  What would put you off becoming involved in a scheme like this? |
| 10.Optimism | How valuable, or not do you feel a scheme like this might be? |
| 11.Environmental context and resources | Can you tell me about the practicalities of what might be required for this scheme to work in practice? Such as resources? |
| 12.Social influences | Can you tell me about what support structures you feel already exist if patients/relatives wish to raise concerns? |
| 13.Emotion | How might the existence of a scheme like this affect you? Can you describe any fears/concerns that you might have regarding patients/relatives being involved in calling for help? |
| 14. Behavioural regulation | Can you describe what might encourage you to becoming more involved in a scheme like this? What might put you off? |
| Prompts | Could you tell me about …  How do you feel about …  What does it mean …  What do you think …  Can you tell me more about …  How do you see … |

*Adapted from Interview schedules by Rainey et al (2015) and Gill et al (2018) and is based on the Theoretical Domains Framework (14 Domains) (Atkins et al 2017; Cane et al 2012)*
