# Supplementary material for: Patients', Relatives' and Healthcare Professionals' Experiences and Perceptions of Raising Concerns About Acute Deterioration: A Qualitative Case Study
Source: J Adv Nurs. 2026 Jul 8;82(12):10379–89. doi: 10.1111/jan.70669 (PMC13752666; doi:10.1111/jan.70669)
Supplement: Supplementary file 2 [file JAN-82-10379-s002.docx]

**Supplement 2**

**Data Analysis for Stakeholder Interviews**

| **Theme** | **Sub-themes** | **Codes** | **Quotes** |
| --- | --- | --- | --- |
| Experiences of Deterioration | Knowing the patient  Being heard | Know self/loved one best, Intuition, ‘You just know’, Previous experience of normal contributes to insight, Awareness of physical symptoms, Awareness of behavioural changes, Awareness of ‘something not right’, Patients/relatives lack of awareness of changes, Inability to appreciate deterioration.  Feeling heard, Feeling not listened to, Feelings/Emotions/consequences associated with being heard/listened to, Feelings/Emotions/consequences associated with not being heard/listened to, Barriers to listening, Facilitators to listening | *We went in one day and he … was trying to grab things. You know the way confused patients do ... And I was saying to the nurses, something is not right here. (Relative 4, A [42])*  *It’s like a mother knowing their child .... Doesn’t matter what the doctor will tell her, oh he’s fine, or she’s fine ... Because in fairness, the doctors … they are only getting to know him. Whereas I know him for years. They don’t. (Relative 1, B [51])*  *Take paeds, the relationship from parent to child is much closer ... They are the extension of the child ... The relationships between an adult patient and their family tend to be much more webbed. (Night Manager, B [22])*  *He was sleeping a lot and ... and I did tell someone. I said he doesn’t seem himself today. And they said, no, it’s just his condition.* *And I said no, … it’s not. He is not himself. Then the following day I got a call … that he was needing ICU. And I said ... I said that yesterday. But ... I did feel that when I did raise it … like when everything is right on their printouts … then there’s nothing wrong. (Relative 1, A [39])*  *I felt that I wasn’t being listened to and that nobody was acting, either on his behalf or my behalf, quickly enough, in terms of the seriousness of the situation. (Relative 3, A [35])*  *I don’t think there’s any formal policy that encourages people to say, you know, if you are worried about someone … Like with paediatrics they have that chart. (Ward nurse 3, FG1, B [28])*  *I think it’s an untapped resource and … we don’t use it enough. You can gain a lot of insight, especially with behavioural changes. And you are not going to be able to pick that up as quickly as a relative … my personal opinion would be … maybe we are missing a trick … I think some people are potentially slipping through the net that we could catch if we were to listen. (Critical Care Doctor, A [02])* |
| Challenges in calling for help | Knowledge/ Skills deficits  Personal and role conflict factor  Resource/organisational deficits | Lack of knowledge to recognise deterioration, Lack of deterioration assessment skills, Unaware of how to raise concern, Lack of knowledge of who to escalate to, Not knowing how to get help for deterioration concerns, Uncertainty regarding what to escalate  Personality characteristics, Lack of experience, Beliefs about patient/relative escalation role, Belief ‘staff know best’, Concerns about ‘stepping on toes’, Personal relations between patients/family and staff  Limited resources, Short staffed, Limited Critical Care Outreach Team capacity, Existing escalation support structures limited, Restricted visiting, Complaints policy but no concerns policy, Lack of escalation structure, Lack of formal policy for escalation of deterioration concerns, Deviation from traditional organisational escalation norms, Cultural barriers to escalation | *It was utterly frightening. And demoralising. You feel helpless. You feel that … are people actually listening to me? And who do I go to? Because I did say, I know my mum better than anybody else. And this isn’t her. But it wasn’t until I said the two magic words. I am a nurse did they begin to listen to me. (Ward Nurse 2, FG2, B [30])*  *But it’s just you don’t want to be stepping on someone else’s toes almost. Do you know what I am saying? (Relative 1, B [51])*  *I’d have no problem talking to the doctor and saying what I think. But I know my mum, she’d be afraid to go up and talk to a nurse in case (Relative 4, B [54])*  *People’s expectations now include being listened to and voicing an opinion ... Which is no bad thing, to be truthful, when it comes down to it. (Night Manager, A [05])*  *Well, I would very much believe in advocating … But like a lot of people I would be intimidated in a hospital as well, I think. (Relative 3, B [53])*  *I have great respect for nurses and I think they do a marvellous job ... But I don’t think they have the time … The resources aren’t there. I don’t want to add to their already busy workload (Relative 1, A [39])*  *With the best will in the world, when we are busy … we struggle to listen carefully and maybe pick up on what can be quite subtle expressions of concern. (Critical Care Doctor, A [02])*  *Sometimes you feel they can be afraid to call ... Oh, I’d better not bother the doctor/nurse, she looks busy. (Ward Manager, B [20])* |
| Ways to support | Signposting  Enhancing engagement | Need for guidance on who to go to, Outline what to look out for to detect acute deterioration, Outline how to get help, Provide education on role.  Enhance existing HCP/Patient/Relative communication channels, Record patient/family concern on EWS chart, Review healthcare professional availability to address concerns, Review visiting policies, Educate staff on patient/relative involvement in escalation, Escalation route outside own care team. | *I don’t think it would hurt to have a leaflet to let you know what to do if you think the patient is getting worse. Because sometimes you sit and go, am I getting worse? Or, should I get the nurse? (Patient 4, A [36])*  *I think that would be very welcome. Even just to reassure you … to listen to you. To voice any possible concerns. Or maybe just to explain what is happening ... I mean anything that would ease a relative’s mind or ease a patient. And that’s the process there that you can follow. (Relative 2, B [52])*  *I think it might be useful, for instance, to have posters/pamphlets in relation to the deteriorating patient ... Yes. I could imagine really around if your loved one appears not themselves, confused, agitated, drowsy, it would help you feel free to discuss with a staff member … (Critical Care Doctor, B [19])*  *I am not a hundred percent sure how that would work if a family member was able to do that. So, I think that it has to be someone who is trained and who understands what the criteria are, to call them. So, I’m not sure whether or not it’s a good idea. (Critical Care Manager, A [01])*  *Because … you know, I worry about going home at weekends and on bank holidays … hoping that if there are deteriorations that they are acted on promptly. So, knowing that there was a very easy way to access extra help or higher-level assistance would be reassuring from your patient safety point of view. (Ward Doctor, A [04])*  *I am thinking of the PEW system, which is the paediatric early warning. So, they purposely have a little scoring on that for the relatives’ outlook. Their input on how they feel that their child is. So, I’d say that system probably works really good. We don’t really have it (in adults). (Ward Nurse 2, FG1, B [27])* |
